# Supplementary figures and images for: Preliminary characterization of Plasmodium vivax sporozoite antigens as pre-erythrocytic vaccine candidates
Source: PLoS Negl Trop Dis. 2023 Sep 13;17(9):e0011598. doi: 10.1371/journal.pntd.0011598 (PMC10519608; doi:10.1371/journal.pntd.0011598)

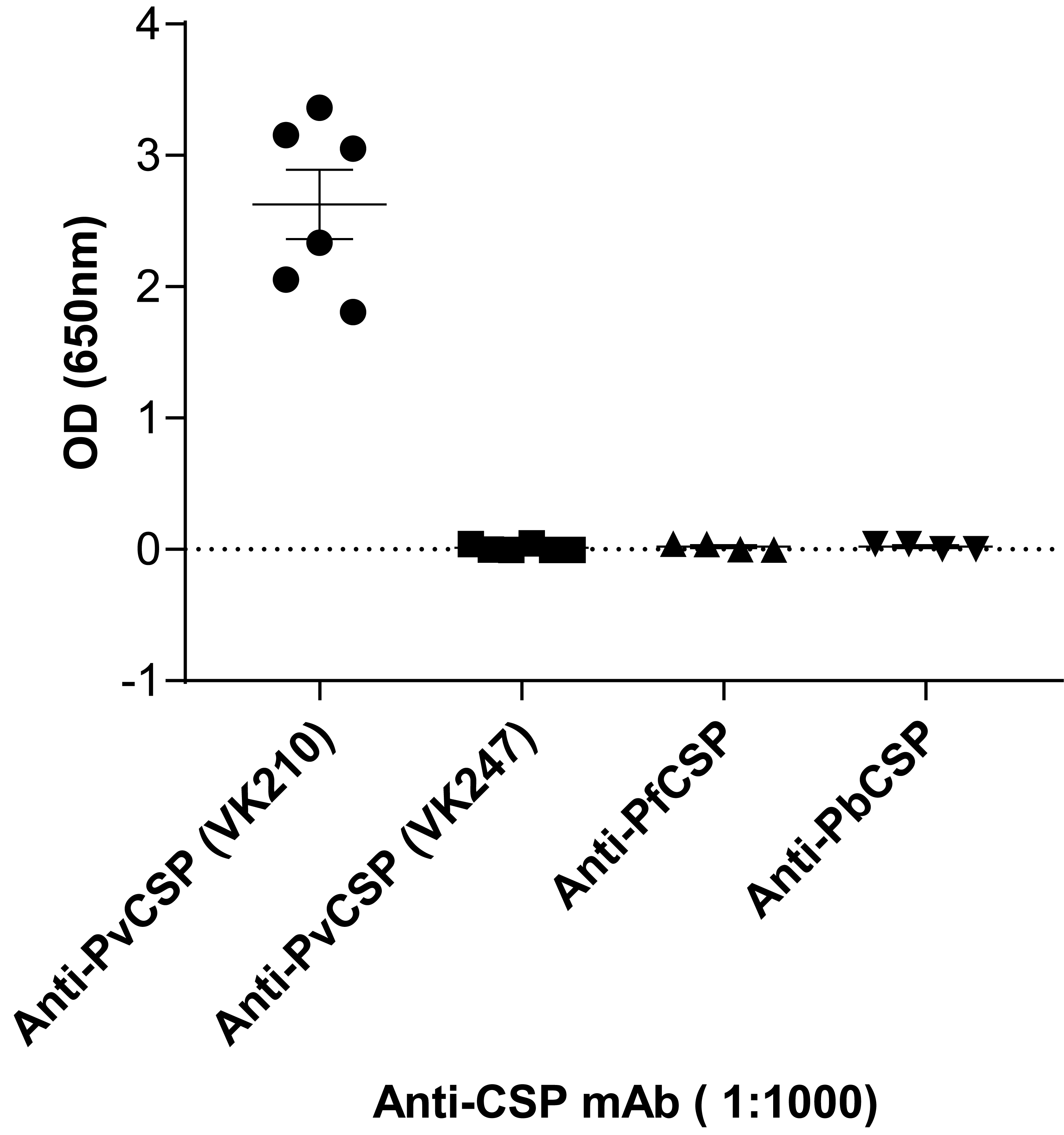

Supplement: S1 Fig — Raw OD values at 650 nm of sporozoite lysate against mAbs (1 μg/ml) specific to the repeat region of CSP for P. vivax VK210, P. vivax VK247, P. falciparum and P. berghei. Error bars indicate s.e.m. (TIF) [file pntd.0011598.s001.tif]

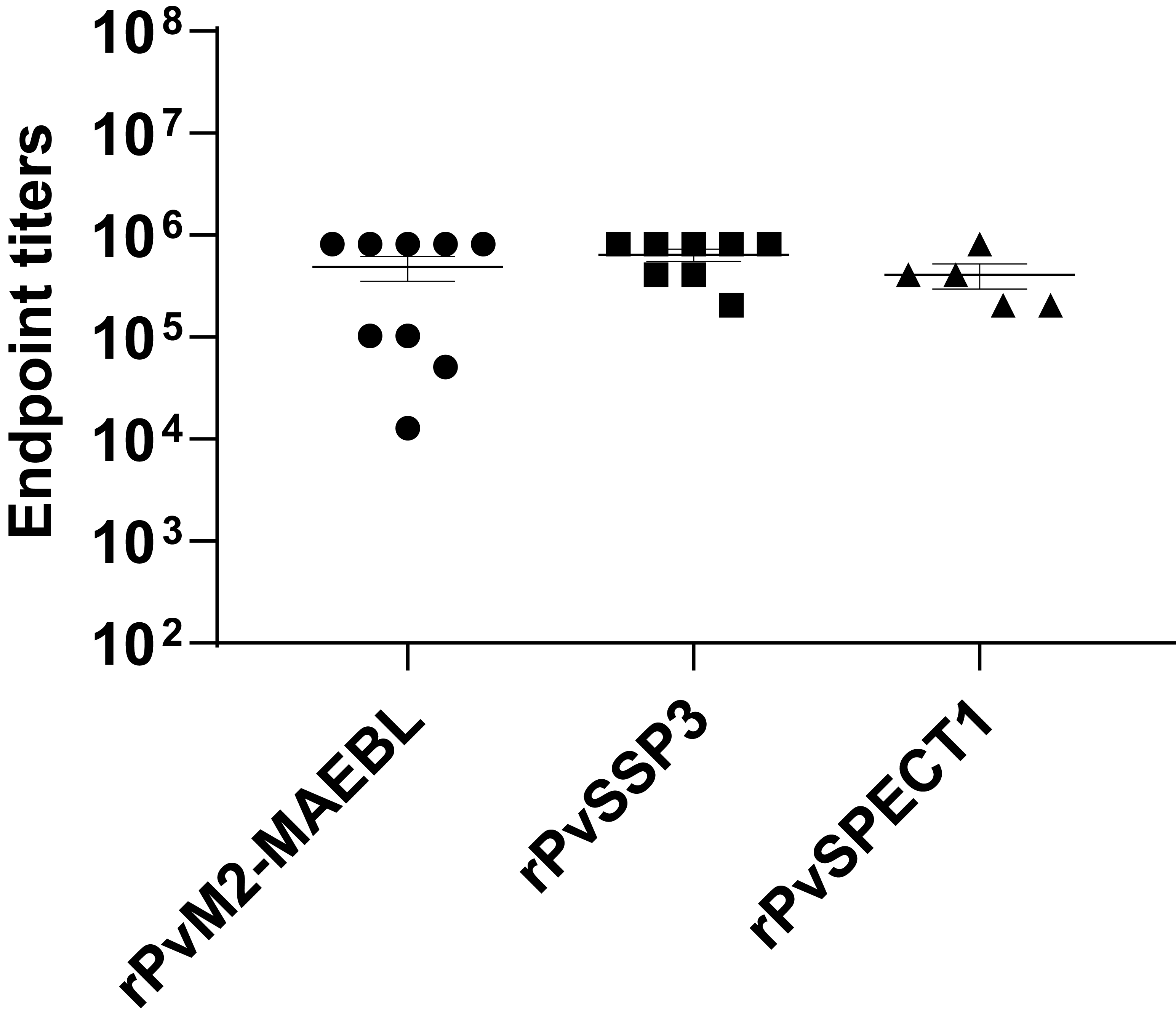

Supplement: S2 Fig — (TIF) [file pntd.0011598.s002.tif]

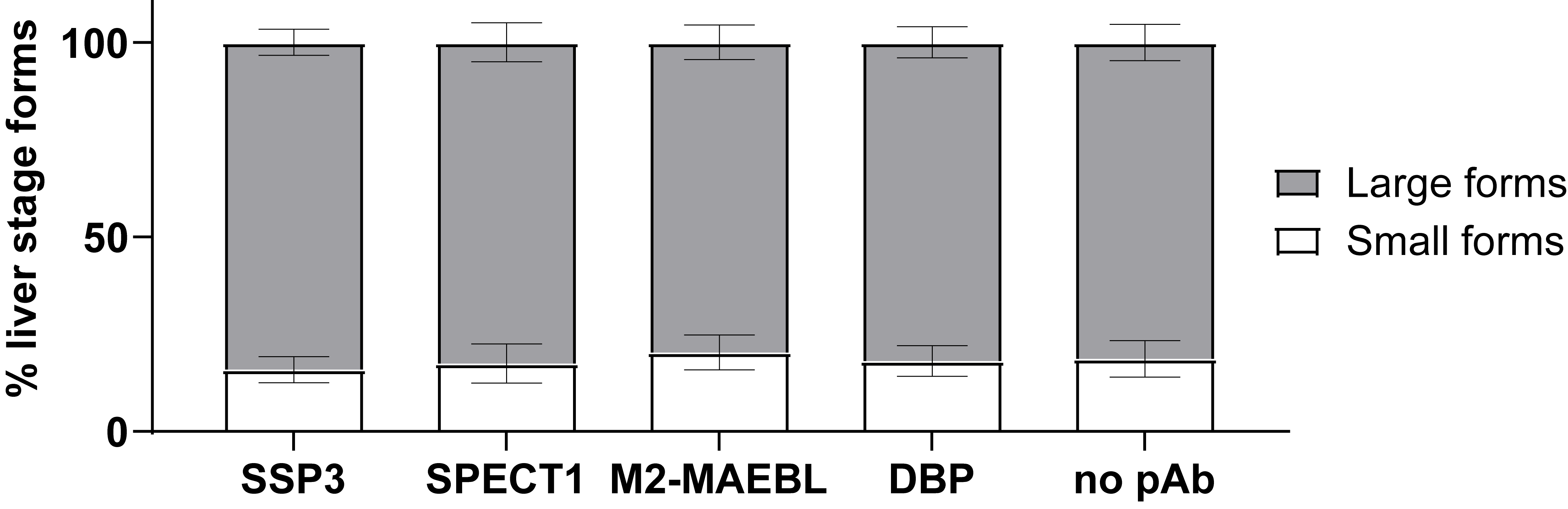

Supplement: S3 Fig — (TIF) [file pntd.0011598.s003.tif]
