# Supplementary material for: Preliminary characterization of Plasmodium vivax sporozoite antigens as pre-erythrocytic vaccine candidates
Source: PLoS Negl Trop Dis. 2023 Sep 13;17(9):e0011598. doi: 10.1371/journal.pntd.0011598 (PMC10519608; doi:10.1371/journal.pntd.0011598)
Supplement: S1 Table — (DOCX) [file pntd.0011598.s004.docx]

| Characteristics | *P. vivax* patients | Healthy subject |
| --- | --- | --- |
| Total Number | 52 | 30 |
| Age (years) | |  |
| Median (Q1, Q3) | 26 (18, 38.5) | 30.5 (24.0, 33.7) |
| Gender | |  |
| Male | 65.4% (34/52) | 50% (15/30) |
| Female | 34.6% (18/52) | 50% (15/30) |
| Nationality | |  |
| Thai | 78.8% (41/52) | 100% (30/30) |
| Myanmar | 21.2% (11/52) | 0 |
| No. of prior infection | |  |
| 0 | 52 | 0 |
| 1 | 0 | 0 |
| >1 | 0 | 0 |
| No. of recorded  re-infections | 0 | 0 |
| Parasitemia (parasite/µL) | | |
| Mean ± SD  (Range) | 4423.51 ± 2690.36  (379.76 - 11608.19) | 0 |
