# Supplementary material for: Preliminary characterization of Plasmodium vivax sporozoite antigens as pre-erythrocytic vaccine candidates
Source: PLoS Negl Trop Dis. 2023 Sep 13;17(9):e0011598. doi: 10.1371/journal.pntd.0011598 (PMC10519608; doi:10.1371/journal.pntd.0011598)
Supplement: S1 Dataset — (DOCX) [file pntd.0011598.s005.docx]

Highlighted sequence indicates recombinant sequence expressed

>PVP01_0948400_M2-MAEBL

MTLYGFLALVAFSCICKTKRIENPQKEFMDRFDIAKNHVDISWSTNGVLGKGDYKYDIDDEKNEYSKLITKNISGTCPNQNIHGMYKGSCPDYGKTFSMDIYRDEYNEDFLNEVSFGFLNKKLNLSIEIPVQKSGMAMYQGLFKYCPLDENHSLLIKKEQEYDMCFKRIYRHMQSIRSNKKRTLRNKYLHFGWHGLGGRLGSNMNYPLHDYNPSESHVTRKMRFSGLIKNLSDCSIYSHCMGPCFNKDFDNECFRSLPVVFNHKTKECVILGTHEGSRRRNCLSEYANGFERCFMPIKKETGKEWTYASSFLRPDYEKKCPPRFPLNDTAFGYYNNSTGECKSAVKGDFVSYEMSFKSCIEGLFNYFGRDRKTRRKNFLWGIWVLEDSSKKLNSMDDIGMCSILKKKPSCVLKKKNHYSFTNLTANSFDFEQNVTYPHVEEMLVQGNEVGEFEKLVLEKSEKEIDLIEMDKKRKKARKEGEEAEQREAYRILEKKRKIKMNEQAGLRKKNLNYVFSMQKSDESTHPNEVDSIFRSKGEPISQMLELNQSSRSYLHNPGARGRGRHQISYVNSQTINNRPASQNEAVVNSRLWTNPQAKFMERFDIPRNHIFIDWKKEGKLGEGNFKYDILSNKTAGTAQSLLVDSYNDICPNHSVPGRAQGSCPNYGKAIIVETLEDKRRDMHFNFQFLNEIHTGYMGKRNGRSIELPYDKSGIAMHHGYPTSCPVNTHEEMLFEKMDDYNYHMCKSSVFSTPFSMKEWDPQSRSIKYYGLYGLGGRLGSNISNYGTYGQTIKRGEKRTSNITLPMKNPGVIKNLFDCSIFSYCLGPCIEDTYKNKCFRNLPAYYNHATNECVILGTHEQERIDNCRKEKTDLSKPNCQKLRKTSDSKDWTYVTSFIRPDYEEKCPPRFPLNSKSFGIYDERTGKCRSLVKKKNFIGALNFDACLEYLFRTSPKDFYSSDAGKYWGVWIANESVNKDNMFIVNGECYYVRRKPTCVIHKEDHFSFTSLTTNDIDFYQNLNIEPLEELIGQRNIIGSDKQDDKSNRATARKQLAVDMALNPSDFNISAKHDGSATEEKDIEEEIRVEEARRVEEARRVEEARRVEEARRVEEARRAEAARKAEEARKAEAARKAEAARKAEADRKAEAARKAEADRKAEAAKKAEEARKAEEARKAEEARKAEEARRAEEARKAEDARKAEADRKAEAARKAEEARKAEAAKKAEAARKAEAAKKAEAAKKAEAARKAEEARKAEAARKAEAARKAEADRKAEAARKAEADRKAEAARKAEAARKAEAAKKAEEARKAEAAKKAEAARKAEAAKKAEEARKAEEARKAEAARKAEDARKAEEARKAEEARKAEEARKAEEARKAEEARKAEEARKAEEARKAEKARKAEEARKAEEARKAEEARKAEEARKAEEARKAEDARRAEEARRAEEARRAEEFRQEENAKKAEEAKKRVVHESKKTEGSAAKGVTDSSDDNREESKEFPKHKFNINNISGENGNSESNSNTEAYNKENFEEEVEEAKMRKQVDDQNMGSENPNSNNAPRNYESRDDKLDKNEYIKRDAEKTREEIINLSKKNPCTVDVSSDFCDYMMKNISFGNCSDGERKGLCCSISNYCLKYFDYNSNDYYTCANEEFGQKDYKCFRKSKASNAAYFAGAGIVLILLLVIASKATLGKWFEEATFDEFDEDYEKVHTFAMISKEQTQKAEPSDFSGHLSDK

>PVX_123155_SSP3

MSNLARYFFLLVLLVSPPVRGRGYVCDFSSSKYNLDFDDYYADVTCYHEIGQGDTIGVIIPKYRDGRENTNILTKCFEEVSLNKSGTKPVSIYQIFSSDEIQVSASNSLYNTEYLSSILKIKNAQTNSYIHCVFENRNEKNKEIHKGVAKIAVKNYPIQNENLTNKHVVDLYNQLDLSKDNSNNKYRVTAEPGHILYILGSKLANGKSIYFGKNCPLHFEYIGDIYKHVFPIINEEEVVYDCPMYYDEKEKQISLGNLIVTFEARPPSIKSISKDILKKHIKYDIERKLHAILSGTDYALANMGSMDRVDSADSDHHRYMEQSIGINETQTSNLQSVNLDREHCNNDKCIDLFENSSCSSLCGGGYRLRDGYDVRYDIQSVIPCNHGDCTAEDSVEPLVIFAWTSIVFFCIMIAILIITIYSLLHVSKQKVADPFYNYDSNIKSSDVL

> PVX_092505_SPELD

MTGGQQMGRGSMAPLVVDTLDCVYLRPQPTSTYYYPLGMTWKYVVSSKSTGCFGTTKKYTLTPETYYYPYYYYYVYYTPAESPIVCLSSKKVIKDKKKKKDEDKQELKDESSKEGSEKEEGSKKSSGKKKYEYVEREKVVRTYLPVVEPFYYTSSYYVPRAILFPEF

>PVP01_1212300_SPECT1

MELFTSLLFLLIALRCALSFEERDVLSHVDKFSVSEYIRKDNDADHGKLQATEYFEKIAEDFSDDINAAKEALQNMFLDVEASFEELSDDVAKSVSQYSYDAEEKLNILEGLVNEFVENSKGVIFNSPEEKKKMEKHKFKKMCDIILEKVKTVVELSTINNYRIILKFGKGERKSEVIDKVKNDDNISDELKSELLKYEDVESKDANVSGLINFISPIYDSFEQKLNALIREVSADLGKII
