# Supplementary material for: Preliminary characterization of Plasmodium vivax sporozoite antigens as pre-erythrocytic vaccine candidates
Source: PLoS Negl Trop Dis. 2023 Sep 13;17(9):e0011598. doi: 10.1371/journal.pntd.0011598 (PMC10519608; doi:10.1371/journal.pntd.0011598)
Supplement: Attachment — Submitted filename: cvrltrNicholasJ PloSNTD_20230727.docx [file pntd.0011598.s009.docx]

July 27^th^, 2023

RE: PNTD-D-23-00587

Dear Editor,

We are submitting a revised manuscript “**Preliminary characterization of recombinant *Plasmodium vivax* sporozoite antigens essential for pre-erythrocytic invasion”** for consideration as an Original Research Article for publication in *PLOS Neglected Tropical Diseases*. We provide below our detailed responses to the reviewers’ comments of this manuscript. The manuscript was revised in accordance with the reviewers’ comments.

We appreciate the opportunity to publish in *PLOS Neglected Tropical Diseases* since our study strongly aligns with the mission statement of the journal*.* As highlighted by both reviewers, there is a dearth of research efforts on the liver stage of *Plasmodium vivax*. We hope our preliminary evaluation of *P. vivax* liver-stage vaccine candidates can inspire future research.

Thank you in advance for considering this revised manuscript for publication in *PLOS Neglected Tropical Diseases*.

Sincerely,

John H Adams, PhD

College of Public Health

University of South Florida - Tampa

ORCID [http://orcid.org/0000-0003-3707-7979](https://nam04.safelinks.protection.outlook.com/?url=http%3A%2F%2Forcid.org%2F0000-0003-3707-7979&data=05%7C01%7Cja2%40usf.edu%7Ca89dfa90ba08449a72a508da601899d1%7C741bf7dee2e546df8d6782607df9deaa%7C0%7C0%7C637927956028149177%7CUnknown%7CTWFpbGZsb3d8eyJWIjoiMC4wLjAwMDAiLCJQIjoiV2luMzIiLCJBTiI6Ik1haWwiLCJXVCI6Mn0%3D%7C3000%7C%7C%7C&sdata=opsmPd0BJaBcz9023yfrnjyJ5zadHno8Jcm8tsvRFX4%3D&reserved=0)

**PNTD-D-23-00587 responses to reviewers’ comments**

***Reviewer #1:*** *1. In the discussion, it is stated “Since humoral effector mechanisms can be bolstered by cell-mediated responses as both play a critical role in protection…” S**ystems serology studies are indicating the important contribution of fc-mediated antibody functions, whereby the antibodies are providing critical support for cellular responses, for example. This could be just as important or even more important than the more classical “sporozoite neutralizing” functionality used as the over-arching objective for this study. This work should be mentioned in the discussion.*

We agree with the reviewer that antibody functions beyond classical “neutralization” are critical for protection. We have revised this discussion point accordingly in lines 485 to 486 “Additionally, recent systems serology studies highlighted the crucial role of Fc-effector mechanisms in controlling malaria infections.”

*2. When discussing proteins, it would be helpful to always place a “Pv” or “Pb” before the name or mention the parasite species in the sentence or paragraph, so the reader knows what it being referred to. For example, in the Methods section “Antigen Production”, the manuscript doesn’t specify what parasite this is, although of course Pv is implied. In the corresponding Results section, it does say “P. vivax” in the heading. In line 412 it does not. This would just help smooth the reading experience.*

We thank the reviewer for bringing this up. In the revised manuscript, we included species distinction of antigens.

*3. Line 88: R21/Matrix-M is now licensed in two African countries, Ghana and Nigeria.*

Thank you for this update. We added this information in the introduction accordingly. Lines 88 to 91 in the revised manuscript.

*4. Line 104: suggest “in hepatocytes” rather than “in the liver”*

Corrected accordingly. Line 102 in the revised manuscript.

*5. Table 1, last entry – To my understanding, the Al-Nihmi reference showed that a parasite that arrests during liver stage immunity (based on knock out of SPELD) shows that (a) SPELD is needed for liver stage development and (b) sporozoites that arrest during liver stage development can induce protective immunity, which is of course not a new finding. I don’t think it shows that SPELD itself is protective. Perhaps this should be reworded as something like “pbspeld ko sporozoites arrest development during the early liver stages” emphasizing the importance of the antigen rather the fact that sporozoites are a protective immunogen which is irrelevant to the manuscript.*

Thank you for this comment. This has been corrected in Table 1.

*6. Figure 3a: I’m thinking it would be better to plot all the values for non-immunized mouse sera as individual data points in their own cloud rather than drawing the dotted line. It doesn’t look as if there is any difference between MAEBL-immunized mice and non-immunized mice. Also suggest that you use the technique of horizontal brackets at the top indicating the p-value for various comparison among the (it would then be four) clusters of data points (this technique is used in Figure 4). Also, if you are wishing to define seroconversion (dotted line), there should be some definition such as x times the median of controls or x standard deviations above controls or something similar specified in the figure legend.*

Figure 3a has been revised accordingly.

*7. Line 455: there is an extraneous “if” that can be omitted.*

Corrected accordingly. Line 456 in the revised manuscript.

*8. The manuscript mentions in several places that one approach to developing an effective vaccine would be to combine antigens. Were the antisera from these antigens looked at in combination?*

For this manuscript, the antisera were not tested in combination. Testing combinations of antisera will be included in future studies.

**Reviewer #2:**

**Major Critiques**

*1. Thank you for the methods section. This will be an asset to the community.*

We appreciate the comment.

*2. Can the authors please show more than a single sporozoite for the IFAs, please? This would make their conclusions about protein localization stronger.*

In the revised manuscript, we included additional sporozoite IFAs for each antiserum in Figures 3b, 3c, and 3d.

*3. There are serious issues with the ILSDA data.*

*a. First, how inhibition is quantified is not described clearly.*

In the manuscript methods section, the description of the calculation to quantify inhibition was revised for clarity.

*Second, description of each readout is obtuse with “inhibition”, “reduced liver stage burden”, “liver stage growth and development was significantly attenuated”, “average schizont area”, and “growth attenuation” all used somewhat interchangeably and without specific references to which measure (e.g. reduction in overall liver stages, reduction in schizonts only, reduction in schizont area) is being utilized.*

Two metrics were used in this study to quantify possible parasite inhibition – i) Number of parasites and ii) parasite size (area). As the reviewer suggested, the results section in lines 351 to 358 was revised to improve clarity.

*b. Furthermore, looking at Table S2, it is unclear how any reductions are seen either in schizonts or overall burden. All conditions have numbers of large forms around or greater than the average of the “no pAb” control of 114.*

Thank you for bringing this to our attention, and we apologize for the error and confusion. Since this was a study on *P. vivax* part of the assessment should include potential inhibitory effect against developing schizonts and hypnozoites even if there was not any difference. We revised the data presentation for the distribution of small and large forms on Day 6 for *P. vivax,* converting the table to a more appealing column chart in Figure S3.

*c. The figures seem to make comparisons between test antigen groups and DBPII for Fig 4a which is appropriate while Fig 4b compares to no serum control. The text references differences between no serum control for significance only. However, the text also notes a large impact of the DBPII serum (which should be a control for liver stages) on growth which suggest the results cannot be attributed to anything but the effects of adding mouse serum. Can the authors please clarify these inconsistencies?*

All antisera did decrease schizont growth. However, only mice immunized with PE-specific antigens significantly decreased growth. DBPII sera did not significantly inhibit the invasion of cryopreserved *P. vivax* sporozoites. Therefore, we speculate lines 355 to 359 of the discussion that our observed decrease in size caused by DBPII sera was possible toxicity of the mouse serum.

*d. There also appears to be no positive control using an anti-PvCSP210 mAb as previously published by this group. Why is this?*

Yes, we did not include anti-PvCSP210 mAb (2F2) as a positive control on purpose. Since this study was analyzing immune sera, we limited our study design to include controls for no serum and a non-specific immune serum instead of using the purified monoclonal 2F2 (Anti-PvCSP210 mAb). Additional negative controls were not really needed and we have a limited stock of cryopreserved *P. vivax* sporozoites available.

*e. Finally, can the authors expand on why there is no impact on hypnozoites/small forms? This is intriguing and in contrast to published work with anti-CSP mAbs.*

In our preliminary antigen characterization, we did not observe a shift in the proportion of hypnozoites upon treatment with antisera. Vantaux et al., 2022 explored hypnozoite development and determined that donor hepatocyte lots can play an important role in differentiation into hypnozoites and schizont. Our study used a single donor lot, and we could not evaluate a host cell effect. This and the many other possible variables will need to be explored in future studies.

*4. ELISA titers from the immunized mice should be shown even if in supplemental.*

As suggested by the reviewer, we added ELISA titers of immunized mice to the recombinant antigens in supplementary figure 2 of the revised manuscript.

*5. If both MAEBL and SPECT1 are micronemal, why do they have different staining patterns? Micronemal localization is used to justify both the surface staining of SPECT1 and the apical localization of MAEBL.*

The morphology of cryopreserved sporozoites in IFA is less distinct than when directly using freshly dissected sporozoites even though sporozoites infectivity remains high (Roth et al., 2018 Nature Communications). Nonetheless, our IFA images are like our previous report for MAEBL and CSP (Preiser et al., 2004), and the Kariu et al. publication. Our reference to localization is reliant on the prior studies. We are unsure if cryopreservation and thawing introduced this variation in staining patterns with SPECT1 or if there were differences in sporozoite activation status in the different studies. Ultimately, immunoelectron microscopy will be needed to validate localization within the sporozoite.

**Minor Critiques**

*6. Line 94: This sentence is difficult to understand. Is the suggestion that PvCSP in a multivalent (ie with other antigens) would increase immunogenicity? In addition, “poor immunogenicity” cannot be caused by a lack of long-lived responses rather they a lack of long-lived responses are a result/indicator of poor immunogenicity.*

In the revised manuscript, this sentence (lines 93 to 94) was modified as follows “Additional studies suggest a lack of long-lived protective responses of PvCSP may be due to poor immunogenicity.”

*7. In Line 112-114, the authors state that “gliding is essential for infectivity” yet cite a publication (REF 25) in which SSP3- sporozoites are gliding deficient yet perfectly capable of infection in vivo. The next references describe parasites with varying levels of gliding with inconsistent effects on infectivity. If there is a more specific scenario in which gliding is essential, could the authors please specify such that the biological importance of in vitro gliding can be better interpreted by the uninitiated reader?*

We updated the language accordingly. The term “is essential for” was replaced with “plays an important role in”. Additionally, we cited a study more relevant to the motility phenotype in Line 111.

*8. Similarly, the terms “gliding”, “invasion” and “infection” are used ambiguously. These are nuanced parts of the complicated parasite life cycle and therefore should be used with more precision and consistency.*

We thank the reviewer for bringing this to our attention. The revised the manuscript now reflects more accurate usage of the terms - “gliding”, “invasion” and “infection”.

*9. Table I, column 1, “essentiality to invasion” is a bit misleading and would be better served by “putative function” or similar. Likewise, “Association with infectivity” appears to rather be “evidence as protective immune target”*

In the revised manuscript, Table 1 was changed as recommended by the reviewer. The phrases “essentiality to invasion” and “Association with infectivity” were changed to “Putative function” and “Evidence supporting immune targeting”, respectively.

*10. This is a nitpicky nuance, but since the sporozoites being used are fixed prior to staining, this is not exactly “native”.*

We edited the result section concerning the sporozoite IFA and ELISA to clarify this.

*11. Why were different t-tests used in Fig. 5? Related to this, based on the binary classification of responders vs. non-responders (using RI of 1) and the Results section which describes results in terms of the percentage responding, a proportional statistical test is likely more appropriate rather than a t-test which would be appropriate for a titer-based comparison.*

Thank you for this observation. We revised Fig. 5 accordingly.

*12. The last line (507-509) should read something like: “the further evaluation of the immunogenicity…could aid in the development”. Given that the functionality data of this manuscript are thin and do not include any combinatorial effects this should remain a speculative statement.*

As suggested, we modified the concluding statement in the revised manuscript (lines 508 to 511) to “Ultimately, the further evaluation of immunogenicity and protective efficacy of each of these antigens alone or in combination could aid in the development of an effective vaccine against *P. vivax*.”

Manuscript ID: PNTD-D-23-00587R1 – additional corrections response to editorial office email 31-JUL-23 from *Nithya Venkatesan.*

*Thank you for submitting your paper, "****Preliminary characterization of Plasmodium vivax sporozoite antigens as pre-erythrocytic vaccine candidates****", to PLOS Neglected Tropical Diseases. We have checked over your submission and, before we can assign your manuscript to the Editor, we ask that you first please make the following modifications:

1) Please amend your detailed Financial Disclosure statement. This is published with the article, therefore should be completed in full sentences, and contain the exact wording you wish to be published.*

*i). State the initials, alongside each funding source, of each author to receive each grant.*

*ii). State what role the funders took in the study. If the funders had no role in your study, please state: “The funders had no role in study design, data collection and analysis, decision to publish, or preparation of the manuscript.”*

*iii). If any authors received a salary from any of your funders, please state which authors and which funders.*

**Funding**

This study was supported by the National Institutes of Health grants U01AI155361 to JHA and U19AI089672 to LC. U01AI155361 provided partial salary support to JHA, JN, SLD, PT, SKK, PAS, SJB, PC, and FBN. U19AI089672 provided partial support to LC and AMB. The funders had no role in study design, data collection and analysis, decision to publish, or preparation of the manuscript.

*2)  We noticed that you used “data not shown” in the manuscript. We do not allow these references, as the PLOS data access policy requires that all data be either published with the manuscript or made available in a publicly accessible database. Please amend the supplementary material to include the referenced data or remove the references.*

Thank you for bringing this to our attention the nested PCR reference has been updated according to PLOS data access policy.

*3) We do not publish any copyright or trademark symbols that usually accompany proprietary names, eg (R), (C), or TM  (e.g. next to drug or reagent names). Please remove all instances of trademark/copyright symbols throughout the text, including Star™ on line no 147,SeeBlue™ on line no 167,ToxinEraser™ on line no 170,ProLong™ on line no 237,InVitroGro™ on line no 258,Shot® on line no 147,TiterMax® on line no 198,199.*

We removed all references to trademarked symbols from the manuscript.

*4) We note that your Main Figures, Table S1, Figure S1" files are duplicated on your submission. Please remove any unnecessary or old files from your revision, and make sure that only those relevant to the current version of the manuscript are included.*

Thank you, these ‘re-appeared’ during the process of uploading the new files and we removed duplicated figures.

*5) Please upload all main figures (Revised Figure 5v2_20230728.pdf) as separate Figure files in .tif or .eps format. For more information about how to convert and format your figure files please see our guidelines:*

All figures are in tif file format.

6*) We have noticed that you have included SI Legends but "Table S2"legend is missing in the file. Please add a full list of legends for your Supporting Information file after the references list.*

Table S2 was replaced in the revised manuscript to the supplemental file as Figure S3. The manuscript has been updated accordingly.

*7) Thank you for stating "Protocols for human subjects were approved by the Ethics Committee on Human Rights Related to Human Experimentation, Mahidol University Thailand [MU-IRB 2012/079.2408].Informed consent was obtained from all participants".*

*Please state "whether written/verbal consent was obtained from the Participants".*

Written informed consent was obtained from all participants. This statement was added to the Ethics statement.
